# Editorial: Cellular and host immune responses in the context of dual viral infection

**DOI:** 10.3389/fimmu.2025.1641084

**Published:** 2025-06-20

**Authors:** Mohamed Mahdi, Vartika Srivastava

**Affiliations:** ^1^ Department of Biochemistry and Molecular Biology, Faculty of Medicine, University of Debrecen, Debrecen, Hungary; ^2^ Department of Infectology, Faculty of Medicine, University of Debrecen, Debrecen, Hungary; ^3^ Department of Inflammation and Immunity, Lerner Research Institute, Cleveland Clinic, Cleveland, OH, United States; ^4^ Clinical Microbiology and Infectious Diseases, Faculty of Health Sciences, School of Pathology, University of the Witwatersrand, Johannesburg, South Africa

**Keywords:** co-infection, superinfection, HIV, adenovirus (Ad), Zika (ZIKV), HCV (hepatitis C), HBV - hepatitis B virus

Co-infection refers to the concurrent invasion of a host by multiple distinct pathogens, a phenomenon frequently observed in clinical settings, and one that poses substantial challenges for accurate diagnosis and effective patient management. To improve therapeutic strategies and clinical outcomes, it is essential to investigate the temporal dynamics of co-infecting pathogens, understand the molecular mechanisms governing their interactions, and assess their collective impact on the host cellular pathways.

In this Research Topic, several studies provide novel insights into the complex immune dynamics involved in viral and bacterial co-infections. For example, a study by Nettere et al. focuses on the role of invariant natural killer T (iNKT) cells in viral infections; a subject that has remained somewhat elusive, largely due to their low frequency in peripheral blood compared to their enrichment in tissues such as the liver. These cells rapidly recognize glycolipid antigens presented by CD1d molecules and initiate a potent, cytokine-driven cytotoxic response. Despite their abundance in the liver, the precise function of iNKT cells in hepatic immune responses remains incompletely understood. While studies in murine models of hepatitis C virus (HCV) infection suggest a protective role, iNKT cells have also been implicated in chronic liver inflammation. Notably, due to their expression of CD4, iNKT cells can serve as direct targets for HIV infection. Given their proposed involvement in liver regeneration following hepatotropic viral infections, it remains unclear how HIV co-infection might alter iNKT cell function. In their study, Nettere et al. address this question by examining peripheral blood iNKT cells in individuals co-infected with HIV and HCV. The authors report that HIV impaires the expansion of iNKT cells following T-cell receptor (TCR) stimulation, indicative of a state of anergy. This functional impairment may compromise antiviral immunity and delay liver repair in the context of co-infection.

Another contribution by Tisoncik-Go et al. explores the impact of SIV-induced immunosuppression on Zika virus (ZIKV) co-infection using a pigtail macaque model. Their findings demonstrate that SIV alters both innate and adaptive immune responses, resulting in a hyperinflammatory state, prolonged ZIKV viremia, and persistent viral presence in gastrointestinal tissues. These results suggest that HIV/ZIKV co-infection in humans may be associated with prolonged symptomatic periods, delayed viral clearance, and possibly more pronounced gastrointestinal involvement.

Focusing on adenovirus-related pathogenesis, Ma et al. investigated the pathogenicity of different human adenovirus (HAdV) serotypes, specifically serotypes 3 and 7, both being known causes of lower respiratory tract infections in children. Their study found that HAdV-7 is associated with more severe clinical outcomes compared to HAdV-3. Notably, co-infections with parainfluenza virus and mycoplasma pneumoniae were more frequently observed in patients infected with HAdV-7, suggesting a higher susceptibility to co-infections and potentially more complicated disease progression.

Guo et al. investigated genetic polymorphisms influencing the efficacy of pegylated interferon-α (Peg-IFNα) in 124 HBeAg-positive chronic hepatitis B (CHB) patients using the Asian Screening Array. They identified that the G alleles at SNPs rs2278420 and rs6509607 were significantly more frequent in patients who achieved a complete response, including HBeAg loss. Expression quantitative trait loci (eQTL) analysis revealed that ZNF350 expression was elevated in patients with genotypes associated with suboptimal response, suggesting a regulatory role in treatment outcomes. *In vitro* stimulation with IFNα showed that individuals with the AA genotypes at both loci had increased mRNA expression of SOCS3, and for rs6509607, additional immune regulators such as PKR, STAT2, SOCS1, PIAS1, PTPN6, and TRIM8 were upregulated. These findings highlight a genotype-dependent modulation of interferon signaling, potentially through the JAK-STAT pathway, influencing individual responses to Peg-IFNα.

Zaongo et al. present a comprehensive review exploring how antiretroviral therapy (ART) may contribute to the onset of type 1 diabetes mellitus (T1DM) in people living with HIV (PLWH). The authors highlight emerging clinical evidence linking immune reconstitution following ART to the development of T1DM as an autoimmune complication. They provide a detailed overview of the immunopathogenesis of T1DM, including the destruction of pancreatic β-cells, and propose mechanisms by which ART-induced immune changes—such as immune reconstitution inflammatory syndrome (IRIS), chronic immune activation, molecular mimicry, and regulatory T-cell dysfunction—may trigger or exacerbate autoimmunity. The review integrates clinical and immunological insights to support the development of targeted prevention and therapeutic strategies for at-risk PLWH. This work adds to the broader discussion on HIV-related comorbidities in the context of viral coinfections and immune dysregulation, emphasizing how HIV treatment itself can intersect with autoimmune and inflammatory pathways.

Wu et al. on the other hand, provide a comprehensive review of recent advances in human papillomavirus (HPV) research, focusing on its intricate interactions with the host immune system and co-infecting agents. The authors explore the role of the microbiome in HPV-associated carcinogenesis and detail HPV’s involvement in the development of multiple cancers, including those of the anogenital tract, head and neck, breast, lung, and prostate. The review also addresses current preventive and therapeutic strategies, from vaccination and screening to targeted therapies and immunotherapies. Future research directions are highlighted to improve the prevention, detection, and treatment of HPV-related malignancies. By examining how HPV interacts with other pathogens and modulates immune responses, this work contributes to the broader understanding of viral coinfections in immunocompromised populations, including people living with HIV.

In summary, the studies presented in this Research Topic underscore the complexity and clinical relevance of viral infections and co-infections, particularly in immunocompromised populations. These contributions illuminate the multifaceted interplay between host immunity and pathogens. As our understanding of co-infection biology deepens, it becomes increasingly clear that effective diagnosis, treatment, and prevention require an integrated approach; one that accounts for both pathogen dynamics and host factors.

